# Not all the number of skeletal muscle fibers is determined prenatally

**DOI:** 10.1186/s12861-015-0091-8

**Published:** 2015-11-11

**Authors:** Mingsen Li, Xingyu Zhou, Yaosheng Chen, Yaping Nie, Huaxing Huang, Hu Chen, Delin Mo

**Affiliations:** State Key Laboratory of Biocontrol, School of Life Sciences, Sun Yat-Sen University, Beisan Road, Guangzhou, 510006 China

**Keywords:** Lineage-tracing system, Myofiber number, Development time frames

## Abstract

**Background:**

The investigation of skeletal muscle development is of importance in stock farming and biomedicine. It is still ambiguous that whether animals are born with the full set of skeletal muscle fibers or if the number of myofibers continues to increase postnatally.

**Results:**

Here, an inducible lineage-tracing system was employed to monitor the changes of myofiber number in various skeletal muscles during development. We confirm that the total myofiber number of longissimus dorsi, gastrocnemius and rectus femoris is determined prenatally. However, tibialis anterior and extensor digitorum longus have a different development pattern, and their myofiber number still increases in the first postnatal week and then remains stable afterwards.

**Conclusions:**

Our results highlight different development time frames of anatomically distinct skeletal muscles.

## Background

Skeletal muscle, as the locomotive and metabolic organ of vertebrate, is mainly composed of thousands of contractile myofibers that are multinucleated and formed from myoblasts [1]. The formation process of functional myofibers, which is termed myogenesis, occurs during embryonic development, postnatal growth or regeneration [1]. Understanding skeletal muscle development is meaningful for agricultural animal production, human health and therapy of muscle associated diseases.

The time when the myofiber number is determined in mammal animals has not been well-established till now. Numerous studies suggested that the total skeletal muscle fiber number is determined during embryogenesis in various species, including mice [2, 3], rat [4], pig [5], cattle [6] and chicken [7]. Postnatal growth of skeletal muscle is mainly realized through increases in length and girth of the myofibers, but not by increase in the number of muscle fibers [8]. Specifically, it was reported that there is no significant increase in the fiber number of extensor digitorum longus (EDL), tibialis anterior (TA), rectus femoris (RF), longissimus dorsi (LD), Soleus (SOL), biceps brachii and Sternomastoideus in postnatal mice [2, 3]. Rosenblatt and his colleagues also suggested that the fiber number of rat EDL is determined before birth [9]. However, some studies demonstrated that the fiber number of mouse SOL, TA and gastrocnemius (GA) still increases after birth [10, 11]. This inconsistence was quite likely to be attributed to the inaccurate myofiber counting from histological cross sections [12], which was unable to monitor changes of myofiber number from prenatal to postnatal period in the same individual. Notably, the deviation of comparison among individuals cannot be excluded. Furthermore, inaccuracies arise when muscles are of the multipennate type [13, 14] or when myofibers terminate intrafascicularly [15, 16]. Thus, the technical problems resulted in this inconsistency.

The Cre-loxP system is a sophisticated tool for reporter model as well as conditional gene modification in genetically-engineered mice [17–19]. Cre is a molecular scissor that catalyzes recombination between two loxP sites [20]. The Tet-On system is an operon model with a reverse tetracycline-controlled transactivator (rtTA) and a tetracycline-responsive regulatory element (TRE) promoter [21]. RtTA can specifically bind to TRE and subsequently activate the expression of downstream gene in the presence of tetracycline, or its analog doxycycline [22]. Thus, the combination of Cre-loxP technology and Tet-On system is a robust strategy for spatial and temporal genetic operation [21, 23].

In our current study, we applied an inducible Cre-loxP labeling technology to trace the formation of myofibers in various mouse skeletal muscles during development. It is demonstrated that anatomically distinct skeletal muscles show different development time frames. In summary, we answered the traditional question outlined above by using a more superior and compelling technology through this investigation.

## Results and discussion

### The transgenic model for tracing the formation of skeletal muscle fibers

**Fig. 1:**
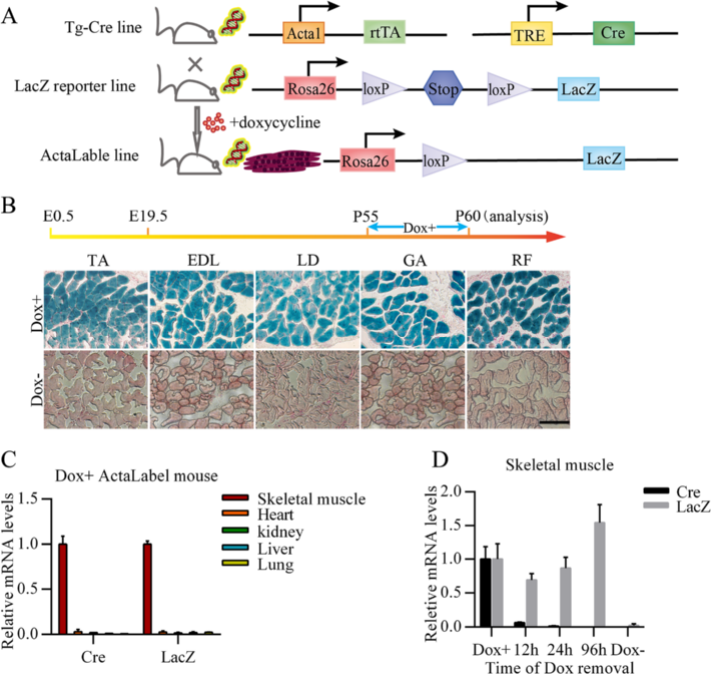
A robust transgenic model for tracing myogenesis. **a** Schematic of doxycylcline-inducible tracing system for perpetual labeling of skeletal muscle myocytes. In ActaLable mice, rtTA is expressed only in skeletal muscle myocytes where ACTA1 promoter is activated. Upon the presence of doxycycline, rtTA drives the expression of Cre recombinase. Then the loxP-floxed transcriptional stop cassette is excised and LacZ expression is activated. **b** β-gal staining of representative transections of LD, GA, RF, TA and EDL. Two-month-old male mice were fed with food and water either with or without doxycycline for 5 days before being analyzed. (Scale bar: 200 μm; *n* = 3 mice per group). **c** Detection of transgene expression specialty in ActaLabel mice. 60-day-old ActaLabel mice were induced with Dox for 5 days and then qPCR analysis was performed to examine the expression levels of Cre and LacZ in the indicated tissues. Gapdh was used as internal control. (*n* = 3 littermates per group, Data present as mean ± SD). **d** Detection for washout time of Dox in mice. 60-day-old ActaLabel mice were administrated with Dox with for 7 days, following 12 h, 24 h and 96 h Dox removal respectively. Then qPCR analysis was performed to examine the expression levels of Cre and LacZ in skeletal muscle. Gapdh was used as internal control. (*n* = 3 littermates per group, Data present as mean ± SD)

To better define the development pattern of skeletal muscle fiber number, we used a doxycycline-inducible tracing system that can permanently label all pre-existing myofibers during the desired time period. Two transgenic strains were employed: Tg-Cre and LacZ reporter (Fig. 1a). Tg-Cre line contains two transgenic constructs: cre recombinase under the control of the TRE and rtTA under the control of the human ACTA1 (actin, alpha 1) skeletal muscle promoter (Fig. 1a). The LacZ reporter line contains a targeted mutation that the DNA sequences encoding LacZ with a loxP-flanked STOP cassette upstream were knocked in the Rosa26 site (Fig. 1a). By crossing these two transgenic lines, the triple transgenic offspring called ActaLabel mice are generated (Fig. 1a). In the ActaLabel line exposed to Dox, the STOP sequence is removed and LacZ is expressed only in skeletal muscle myocytes (Fig. 1a). Consequently, myofibers expressing LacZ can be stained blue in the β-galactosidase (β-gal) assay.

It has been well documented that efficient induction can be achieved in various tissues in this triple transgenic strain of various ages, including embryonic period and postnatal growth [21, 22]. In order to verify our model, 8-week-old ActaLabel mice were treated with doxycycline for 5 days before being analyzed. β-gal staining showed that all of the myofibers (TA, EDL, LD, GA, RF) were labeled blue (Fig. 1b), indicating nearly 100 % induction efficiency. As expected, LacZ expression was rarely detected in the counterparts of no Dox-treated ActaLabel mice (Fig. 1b). Cre and LacZ mRNA expressions were also detected in skeletal muscle of Dox-treated ActaLabel mice, but not in heart, liver, lung and kidney (Fig. 1c). Thus, these transgene expressions were doxycycline-dependent and skeletal myocytes-specific.

To test the washout period of doxycycline in mice, ActaLabel mice were fed with doxycycline diet and drinking water for one week and then switched to control diet and water without Dox for three time periods (12 h, 24 h and 96 h). Quantitative PCR (qPCR) analysis showed that Cre expression was slight after 12 h of Dox withdrawal and negligible after 24 h or 96 h Dox removal (Fig. 1d), which was consistent with the previous report [21]. In contrast, LacZ was expressed at the similar level in all groups (Fig. 1d), indicating that upon the stop cassette is removed, LacZ could be expressed continually even in the absence of Dox. Collectively, these observations suggest that our transgenic model is desirable and suitable for labeling myofibers both spatially and temporally.

According to the donor’s description, expression mosaicism between muscle fibers and muscles was detected when the adult mice were treated with Dox in drinking water for 3 days [22]. In our study, we also observed slight expression mosaicism when mice were fed with Dox for 5 days. However, expression mosaicism almost could not be detected when the mice were fed with Dox both in diet and drinking water for 7 days. Therefore, we speculated that expression mosaicism may be caused by the inadequate Dox exposure. We added Dox both into the diet and drinking water and simultaneously extended induction time to ensure more successive and sufficient induction. Moreover, the induction efficiency of Dox in the embryo may be lower by the mother transmission. For these reasons, we treated the pregnant mice with Dox from E0.5 to ensure more adequate and robust induction to avoid expression mosaicism phenomenon. In addition, induction performed at the beginning of embryonic development ensured that all myofibres formed during embryogenesis could be labelled by LacZ. Remarkably, there was no negative effect of the prolonged administration with Dox observed by us and previous reports [21, 24].

### The myofiber number of LD, GA and RF is determined prenatally

**Fig. 2:**
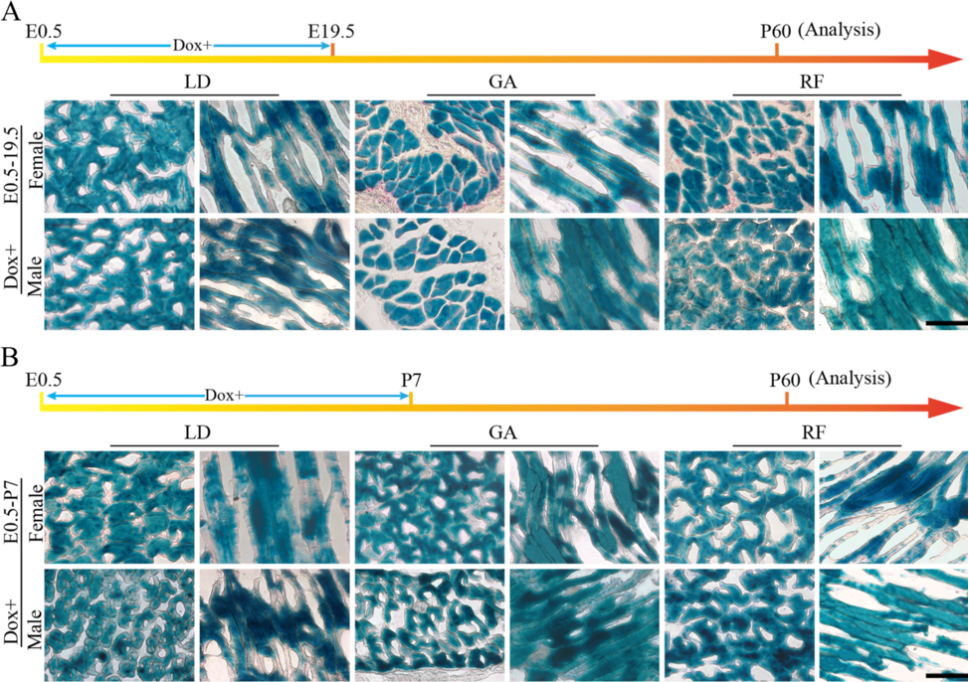
β-gal staining of representative lengthwise sections and transections of LD, GA, and RF. Both female and male ActaLabel littermates were administrated with Dox during the period E0.5–19.5 **a** and E0.5-P7 **b** respectively and analyzed at P60 (Scale bar: 200 μm. *n* = 3 mice per group)

To determine the precise timing of myogenesis during development, ActaLabel mice were continuously exposed to Dox during two periods (E0.5–19.5 and E0.5-P7) respectively and analyzed at postnatal day 60 (P60). All the myofibers pre-formed during the induction period would express LacZ permanently, even after doxycycline withdraw. If the myofibers newly emerge after doxycycline removal, no LacZ signals could be detected in them. In the β-gal assay, it was showed that nearly 100 % LD, GA and RF myofibers were LacZ positive in both female and male adult ActaLabel mice which were administrated with Dox for whole pregnancy (Fig. 2a). Similar results were also observed in adult ActaLabel mice on Dox during E0.5-P7 (Fig. 2b). These observations demonstrate that all of the LD, GA and RF myofibers in mice are formed during embryogenesis, which is gender-independent. Thus, we conclude that the total myofiber number of LD, GA and RF has been determined prenatally in mice, without postnatal increase.

### The myofiber number of TA and EDL is determined within postnatal one week

**Fig. 3:**
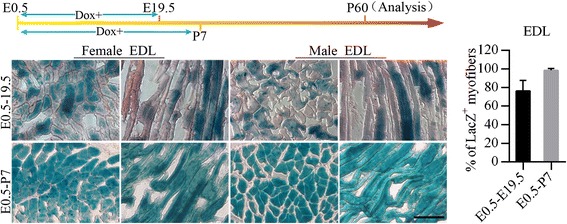
β-gal staining of representative lengthwise sections and transections of EDL. Both female and male ActaLabel mice were induced with Dox during the period E0.5–19.5 and E0.5-P7 respectively and analyzed at P60. Column diagram presents the average percentage of LacZ+ myofibers in the indicated groups. LacZ+ myofibers were counted in 10 fields of muscle sections for each mouse. 3 littermates were analyzed for each treatment. The average showed in the figure was calculated by the data from 3 littermates of each treatment. (Scale bar: 200 μm; *n* = 3 littermates per group, Data present as mean ± SD)

**Fig. 4:**
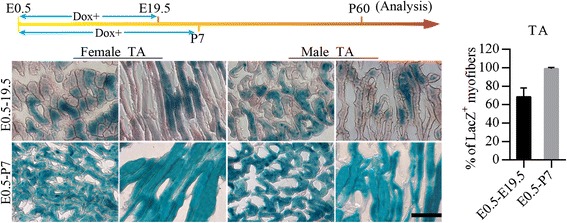
β-gal staining of representative lengthwise sections and transections of TA. Both female and male ActaLabel mice were treated with Dox during the period E0.5–19.5 and E0.5-P7 and analyzed at P60. Column diagram presents the average percentage of LacZ+ myofibers in the indicated groups. LacZ+ myofibers were counted in 10 fields of muscle sections for each mouse. 3 littermates were analyzed for each treatment. The average showed in the figure was calculated by the data from 3 littermates of each treatment (Scale bar: 200 μm; *n* = 3 littermates per group, Data present as mean ± SD)

Intriguingly, unlike LD, GA and RF, TA and EDL displayed a significantly distinctive development pattern. In the 60-day-old mice treated with Dox throughout the whole pregnancy, only around 76 % of the EDL myofibers were LacZ-positive (Fig. 3), whereas nearly 100 % positive β-gal staining was observed after prolonging Dox administration (E0.5-P7) (Fig. 3). These results imply that the LacZ-negative EDL fibers were formed in the postnatal first week and no extra addition of myofibers occurred afterwards. As for TA, a similar pattern was observed in 60-day-old mice which were induced during embryogenesis, with approximately 31 % LacZ-negative fibers were present (Fig. 4). These data indicate that part of the total myofibers in TA emerge after birth. In contrast, when ActaLabel mice were exposed to Dox during the period E0.5-P7, all myofibers in TA were labeled blue at P60 (Fig. 4), demonstrating that there was no increase in the fiber number afterwards. According to these results, we conclude that the total myofiber number of TA and EDL is determined within postnatal one week in both genders.

By using this elegant genetic system, we demonstrate that not all the number of skeletal muscle fibers is determined before birth. TA and EDL continue to add fibers up through the first week of postnatal development. These newly-emerging myofibers were formed from the individual myoblasts instead of the existing myotubes. Thus, it is quite likely that the de novo forming occurs for TA and EDL within the first week of postnatal growth. It has been well documented that postnatal skeletal muscle growth occurs by proliferation, differentiation and fusion of the committed muscle progenitors (mainly satellite cell) [1, 25]. Therefore, we speculated that the newly-formed myofibers within the first postnatal week may be due to the differentiation of muscle progenitors and myocytes fusion.

Remarkably, the visible blue labeling makes the monitor for myofiber number changes more credible and accurate than the traditional counting. Taken together, our data present an overview that anatomically distinct skeletal muscles have different development time windows. The elucidation of kinetics of myofiber number development would provide theoretical basis for meat production and muscle-associated diseases therapy.

## Conclusions

Anatomically distinct skeletal muscles differ in the development time frame. Not all the number of skeletal muscle fibers is determined during the embryonic period.

## Methods

### Mice

Both B6;C3-Tg(ACTA1-rtTA,tetO-cre)102Monk/J (stock number: 012433) and B6.129S4-Gt(ROSA)26Sortm1Sor/J (stock number: 003474) lines were obtained from Jackson Laboratory. Mice were housed in the SPF condition with 12-h dark and 12-h light cycle. After intercrossing of these two lines, the triple transgenic mice were generated, which are indicated as ActaLable mice. Pregnant mice and adult ActaLable mice were induced simultaneously with AIN-93G doxycycline diet (100 mg/kg, RESEARCH DIETS) and drinking water with doxycycline HCl (1 mg/ml in 5 % sucrose), which was refreshed every 3 days. Control groups were fed with AIN-93G control Diet (RESEARCH DIETS, Product # D10012G) and normal drinking water. All animal experiments were approved by the Animal Care and Use Committee of Guangdong Province and carried out in accordance with ethical standards.

### RNA isolation and real-time PCR assay

Total RNA was extracted from mice tissues using TRI Reagent (Sigma) according to manufacturer’s protocol. cDNA was synthesized from 1 μg total RNA by Reverse Transcription Kit (Promega). Real-time PCR assays were performed on the LightCycler 480 system (Roche) using SYBR Green qPCR Mix (Dongsheng Biotech), with GAPDH as an internal control for normalization.

The primers used are listed as follows:

Cre: F: 5′-GGATTAACATTCTCCCACCGT-3′, R: 5′-CGACCAGGTTCGTTCACTCA-3′;

LacZ: F: 5′-ACTATCCCGACCGCCTTACT-3′, R: 5′-CGTCGATATTCAGCCATGTG-3′

GAPDH: F: 5′-CATGGCCTTCCGTGTTCCTA-3′, R: 5′-TGCCTGCTTCACCACCTTCT-3′

### β-Galactosidase Staining of Frozen Sections

The skeletal muscle tissues were isolated from mice and immediately frozen in liquid nitrogen. Then, frozen blocks were embed in O.C.T. compound (Optimal Cutting Temperature compound) and sectioned at 10 microns using Cryostat Microtome. Prior to staining, slides were immediately fixed in fixative solution (0.2 % Glutaraldehyde, 5 mM EGTA, 1 mM MgCl2, in 0.1 M phosphate buffer [pH 7.3]) on ice for 10 min. Then, washing was performed in PBS for 10 min, following in detergent rinse (0.02 % NP-40, 0.01 % Sodium Deoxycholate, and 2 mM MgCl2 in 0.1 M phosphate buffer [pH 7.3]) for 10 min. For the staining procedure, slides were immersed in 1 mg/ml X-gal staining solution (0.02 % NP-40, 0.01 % Sodium Deoxycholate, 5 mM Potassium Ferricyanide, 5 mM Pottassium Ferrocyanide, and 2 mM MgCl2 diluted in 0.1 M phosphate buffer [pH 7.3]) overnight at 37 °C in the dark with slight shaking. Next, slides were post-fixed in 4 % paraformaldehyde (PFA) solution for 10 min before being counter-stained with Nuclear Fast Red. Finally, Gradient alcohol dehydration and mounting were performed. All slides were observed under microscope.

## Abbreviations

EDL: extensor digitorum longus
GA: gastrocnemius
LD: longissimus dorsi
RF: rectus femoris
SOL: Soleus
TA: tibialis anterior
